# Energy Balance and Control of Body Weight: Possible Effects of Meal Timing and Circadian Rhythm Dysregulation

**DOI:** 10.3390/nu13093276

**Published:** 2021-09-19

**Authors:** Alessio Basolo, Susanna Bechi Genzano, Paolo Piaggi, Jonathan Krakoff, Ferruccio Santini

**Affiliations:** 1 Obesity and Lipodystrophy Center, Endocrinology Unit, University Hospital of Pisa, 56124 Pisa, Italy; alessio.basolo@med.unipi.it (A.B.); susanna.bechi.dr@gmail.com (S.B.G.); 2 Department of Information Engineering, University of Pisa, 56126 Pisa, Italy; paolo.piaggi@gmail.com; 3 Obesity and Diabetes Clinical Research Section, Phoenix Epidemiology and Clinical Research Branch, National Institute of Diabetes and Digestive and Kidney Diseases, Phoenix, AZ 85016, USA; jkrakoff@mail.nih.gov

**Keywords:** energy expenditure, food intake, thermic effect of food, chronodisruption

## Abstract

Conservation of the energy equilibrium can be considered a dynamic process and variations of one component (energy intake or energy expenditure) cause biological and/or behavioral compensatory changes in the other part of the system. The interplay between energy demand and caloric intake appears designed to guarantee an adequate food supply in variable life contexts. The circadian rhythm plays a major role in systemic homeostasis by acting as “timekeeper” of the human body, under the control of central and peripheral clocks that regulate many physiological functions such as sleep, hunger and body temperature. Clock-associated biological processes anticipate the daily demands imposed by the environment, being synchronized under ideal physiologic conditions. Factors that interfere with the expected demand, including daily distribution of macronutrients, physical activity and light exposure, may disrupt the physiologic harmony between predicted and actual behavior. Such a desynchronization may favor the development of a wide range of disease-related processes, including obesity and its comorbidities. Evidence has been provided that the main components of 24-h EE may be affected by disruption of the circadian rhythm. The sleep pattern, meal timing and meal composition could mediate these effects. An increased understanding of the crosstalk between disruption of the circadian rhythm and energy balance may shed light on the pathophysiologic mechanisms underlying weight gain, which may eventually lead to design effective strategies to fight the obesity pandemic.

## 1. Introduction

Obesity is a growing public health problem and an ever-increasing global pandemic with individual and general consequences. In 2016, the World Health Organization estimated that 39% of the world population were individuals who were overweight and 13% with obesity [1]. It has been predicted that the prevalence of individuals with obesity will approach 50% of the worldwide population by 2030 [2]. The dysregulation of the two components of the energy balance equation, energy intake (EI) and energy expenditure (EE) leads to weight gain and, thus, to the development of human obesity. Whereas the solution to the obesity “problem” appears as easy as increasing energy expenditure and reducing energy intake, failure of attempts to change individual lifestyle within an obesogenic environment indicates that understanding the complex interactions between genetics, physiology, environmental and social behavior are essential in the control of human energy balance. A major challenge in obesity research is the accurate measurement of energy intake and energy expenditure, to identify and explore causal associations between various components of energy homeostasis, and to accurately define how energy balance is altered in response to different interventions.

In the past years, scientists focused on the emerging role of circadian rhythm in the regulation of energy metabolism. Circadian rhythms are essential in humans to coordinate physiologic functions which are generally tied to the dark/light cycle such as sleep-wake periods, feeding behavior, hormone secretion and metabolism [3]. Evidence for a pathogenic role of disruption of the circadian rhythm exists in several conditions including obesity, diabetes mellitus and psychiatric disorders [4,5]. Recently, it has been shown that eating timing may be more important than macronutrient composition of diet in controlling changes in body weight [6,7].

This review will focus on the effects of circadian rhythm disruption on the regulation of energy balance components (food intake and energy expenditure). It is a narrative review not set to systematically include the entire body of published literature but only the pivotal studies that may provide a broad perspective on the topic.

## 2. The Regulation of Energy Balance: From a Static to a Dynamic Perspective

An essential principle of nutrition and metabolism is that change in body weight is associated with dysregulation between the energy content of consumed food and the energy expended by the human body for basal functions, absorption of ingested food and physical work (or exercise) [8]. Accurate assessment of caloric intake and energy expenditure is difficult to achieve. Indeed, the daily energy intake fluctuates depending on numerous individual and environmental variables. On the other hand, energy expenditure though largely determined by body mass and body composition [9], is itself affected by both physical activity and food consumption. Measurement of these factors is difficult under free-living conditions, but precise measures of energy expenditure can be obtained in a controlled environment, such as a respiratory chamber [10,11]. In humans, the actual amount of energy that is available following food consumption is about 90% of energy ingested, with the rest being lost in feces, urine and skin [12,13].

The many physiological, environmental, genetic and hormonal factors intervening in the regulation of food intake [14,15] and body weight change [16,17,18,19,20] will not be discussed in the current review that will be focused on those related to energy expenditure and circadian rhythms.

The rate of whole-body energy expenditure (EE) varies within a 24-h period and can be accurately and continuously measured inside the respiratory chamber, an open circuit whole room indirect calorimeter [21,22]. By measuring oxygen consumption and carbon dioxide production, the respiratory chamber provides a precise estimation of the energy expended by a human subject every minute over long periods of time. This method is considered the gold standard for the measurement of energy expenditure over 24 h because of the ability to distinguish the daily components of energy expenditure such as resting metabolic rate (RMR), thermic effect of food (TEF) and the energy cost of physical activity (PA).

RMR, the energy needed to ensure vital functions at resting, is the main component of 24-h EE and it can be further classified in the energy expended during sleeping (sleeping metabolic rate; SMR) and the energy expended to be awake excluding the physical activity (cost of arousal) [23]. The main determinant of RMR is fat-free mass as it accounts for ~70% of its variance [9,11,24], whereas fat mass, sex, age, ethnicities and familial traits explain an additional ~15% [25].

The energy cost of PA, defined as the energy consumed during spontaneous and voluntary exercise, is the most variable component of 24-h EE, varying from ~15% in very sedentary subjects to ~50% in very active subjects [26]. Similar to RMR, the energy cost of PA depends on body composition, age, sex, genetic traits, interactions between biochemical, physiological, and brain reward pathways and environmental stimuli [27].

TEF, defined as the increase in energy required to digest, absorb, assimilate and store nutrients after meal ingestion [28], accounts for ~10% of the 24-h EE when subjects are in the energy balance state on a Western diet [29,30], but it shows a large variability among subjects. The components of TEF may be considered to be its obligatory cost, that is, the energy used to metabolize the ingested nutrients, and its facultative component, reflecting the inter-individual response to surplus or restriction in food intake. The obligatory cost depends on the amount and type of macronutrient ingested, accounting for 5–10%, 20–30% and 0–3% of energy from carbohydrates, proteins and fat, respectively [30,31,32,33].

Emerging findings indicate that circadian disruption leads to an increased risk of obesity [34] by altering EE [35,36], which is affected by the timing of sleep, meals and exercise [37]. Furthermore, TEF may be blunted in individuals with obesity during resting conditions, exercise and post-exercise states compared to lean individuals [38], suggesting that obesity might reduce the capacity for thermogenesis in different conditions.

**Figure 1:**
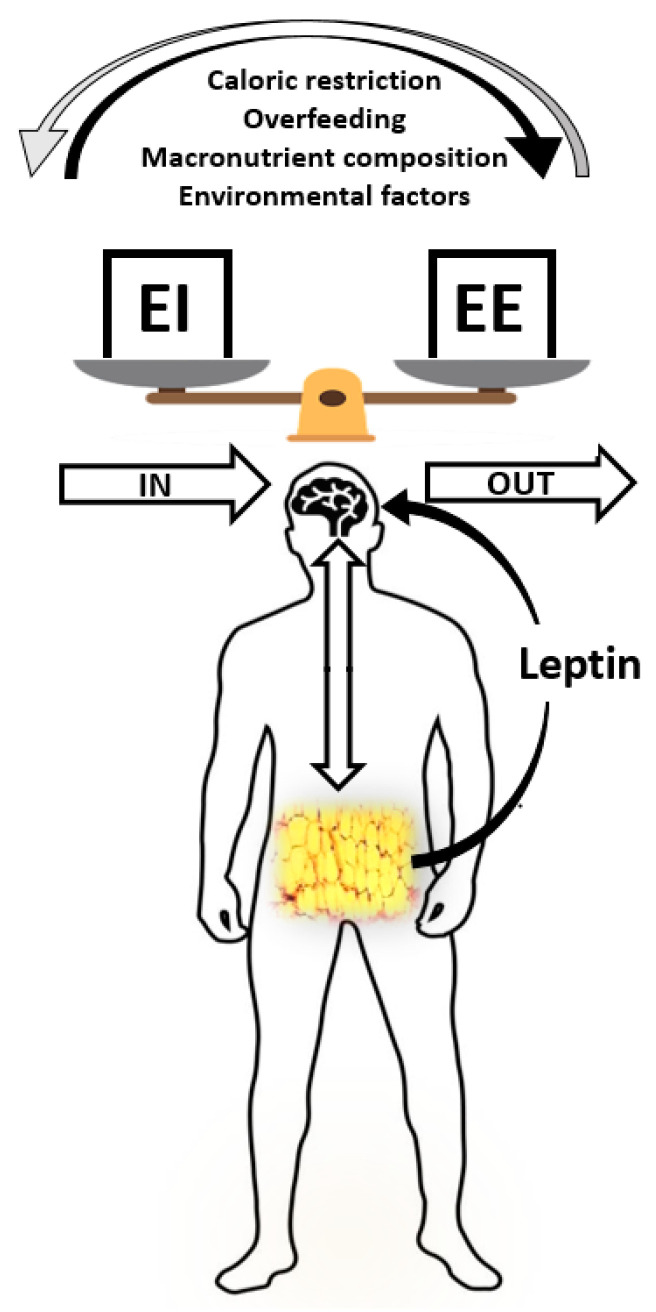
Energy balance and the adipose organ. Disequilibrium in energy balance regulation can be caused by several factors such as caloric restriction, dietary overfeeding, different macronutrient compositions and several environmental perturbing factors, including those causing chronodisruption. The adipose tissue plays an active role in the regulation of energy balance, not only as a site for lipid storage but also being involved in heat generation (in brown adipose tissue) and adipokine secretion. The adipose organ serves as a dynamic energy depot that constantly conveys signals to the central nervous system to communicate the amount of energy stores and to elicit compensatory responses.

An understanding of the physiologic control of energy balance is necessary to design interventions to fight the obesity epidemic worldwide. Commonly, comprehension of the pathogenesis of overweight and obesity is based on a static simple model of energy balance, which suggests that a chronic positive energy balance (i.e., when daily caloric intake persistently exceeds energy expenditure) leads to an increase in body weight and development of overweight/obesity due to the storage of energy surplus as body fat [8,39]. This model assumes that variations in caloric intake and physical activity independently modify the components of energy balance. It must be noted though that energy intake and energy expenditure are dependent on each other and are regulated by a more intricate model, aiming to maintain body weight and body energy stores within a definite range. Conservation of energy equilibrium can be considered a dynamic process and, under ideal physiologic control, variations of one component (energy intake or EE) cause biological and/or behavioral compensatory changes in the other part of the system. According to this model, a reduction in caloric intake induces a correspondent decrease in energy expenditure, while an increase in physical activity is followed by stimulation of hunger to increase caloric intake. In everyday life, such perfect matching between energy intake and energy expenditure is barely achieved, and the adipose tissue serves as a dynamic depot, protecting against unavoidable deviations from the balance equation, and constantly conveying signals to the central nervous system, through the leptin-melanocortin pathway, to communicate the amount of energy stored (Figure 1).

From an evolutionary point of view, the link between the energy demand and caloric intake would guarantee an adequate food intake in variable life contexts, through the mediation of an “energy sensing” mechanism, to preserve life and reproduction. Recent studies have hypothesized that EE plays a role in regulating food intake by conveying peripheral signals to the central nervous system (SCN) to modulate hunger. In support of this hypothesis, it has been demonstrated that resting EE was positively associated with daily energy intake and hunger feeling [40,41] and that 24-h EE directly correlated with ad libitum energy intake [42]. Fat-free mass, the most metabolically active part of the human body, is the main determinant of EE [11], suggesting that it may represent the mediator on food intake. However, it has been recently demonstrated that 24-h EE per se, rather than FFM, along with a preference for carbohydrates over fats as a substrate for oxidation, are independently associated with energy intake [42,43,44].

The efficiency of this feedback mechanism is challenged by clear evidence that most people, under the pressure of an obesogenic environment, increase their body fat stores beyond what is considered physiological and healthy. Lower 24 h EE and RMR predicts an increase in body weight in Native Americans [45] and Caucasians [46], implying a constitutive tendency toward maintenance of a positive energy balance and increasing energy stores. However, in different study cohorts, such as lean Nigerian individuals, higher EE leads to weight gain [47], suggesting that an increase in energy intake might overcompensate the increased energy demand. These apparently contrasting findings indicate that investigating EE in the absence of a measure of energy intake may not provide the full picture of how energy metabolism influences weight gain [48]. When EE and ad libitum intake are available in the same study, a positive deviation from the energy expenditure-food intake relationship should be associated with weight gain. In Native Americans who are overweight a relatively greater food intake, after accounting for 24-h EE, predicted weight gain at a 2-year follow up, suggesting that the individual predisposition to overeat in relation to the metabolic request may indicate the propensity of a subject to gain weight over time [49].

The thrifty phenotype hypothesis encompasses the idea that it was beneficial for bodyweight preservation to conserve energy (e.g., lower metabolic rate) for hunter-gatherer populations. In today’s obesogenic environment, thriftiness is no longer beneficial and would lead to excessive accumulation of energy stores that will never be dissipated.

Assessment of metabolic thriftiness may be better evaluated during energy balance disequilibrium. However, this is difficult to measure in free-living conditions due to requiring careful control of food intake and precise measurement of EE. Under such specifically controlled conditions of positive or negative energy balance, deviations in expected EE responses have been observed. This has led to the identification of EE phenotypes that predispose to weight gain or loss. These phenotypes were characterized based on changes in EE during acute dietary interventions, such as 24 h fasting and 200% overfeeding [50,51,52]. The results of these studies indicate two different phenotypes: (i) “thrifty”, an energy-efficient phenotype characterized by a larger decrease in EE during fasting and smaller increases in EE during overfeeding (ii) “spendthrift”, a phenotype with smaller decreases in EE during fasting and larger increases in EE during overfeeding [51,52,53]. In 14 men, 24-h EE decreased by ~10% from its baseline during 48-h fasting whereas 24-h EE increased by ~10% following a balanced overfeeding diet with 200% of energy needs (50% carb, 30% fat and 20% protein) [51]. A follow-up in 37 healthy subjects confirmed that a greater decrease in EE during fasting was associated with a lesser EE increase during overfeeding diets with varied macronutrient content [52]. Moreover, “thrifty” participants (those with greater EE decrease during fasting) had an increase in body weight at 6-month follow-up [52]. In a highly controlled inpatient setting, in 12 subjects with obesity who underwent 6 weeks of caloric restriction, thrifty individuals displayed a lower rate of weight loss compared to spendthrift subjects [50]. Recently, it has been shown that thrifty individuals had a greater increase in body weight and fat mass following 6 weeks of daily low-protein overfeeding, as compared to spendthrift subjects [54].

The mechanisms involved in the EE responses to dietary intervention in humans have not been fully clarified. It has been hypothesized that body composition [30], core body temperature [55], hormones [18,56,57], brown adipose tissue [58,59] and sympathetic nervous system tone [17,57,60,61] might play a role in regulating the metabolic adaption. Recently, it has been proposed that other factors such as the circadian rhythm might play a role in the regulation of the energy balance, through a change in food timing and thermic effect of food, thus influencing body weight regulation [62,63].

## 3. Circadian Rhythm and Energy Balance

The circadian rhythm plays a major role in systemic homeostasis by acting as the “timekeeper” of the human body and it is regulated by central and peripheral clocks. Clock-associated biological processes anticipate the daily demands imposed by the environment, being synchronized under ideal physiologic conditions [64,65]. The central clock, located in the suprachiasmatic nucleus (SCN) in the anteroventral hypothalamus [3], collects light cues, which are the main stimuli for maintaining 24-h cycles [66]. Importantly, the master clock plays a role in synchronizing behavioral and metabolic rhythms to the light/dark cycles [67,68].

**Figure 2:**
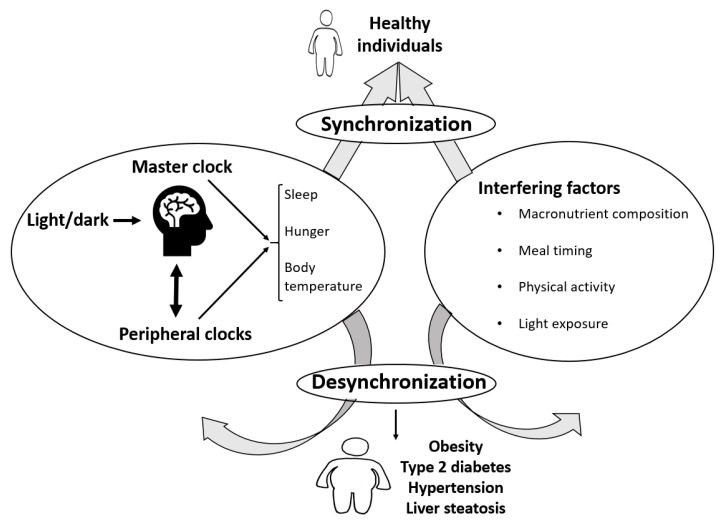
Human circadian rhythm: link between circadian clocks and environmental factors. The central clock and peripheral clocks regulate many physiological functions such as sleep, hunger and control of body temperature. Factors that interfere with the expected demand, including macronutrient composition, meal timing, physical activity and light exposure, may disrupt the physiological harmony between predicted and actual behavior. Such a desynchronization may favor the development of obesity and its complications.

The master clock in the hypothalamus conveys circadian signals to peripheral clocks that are present in several tissues (liver, pancreas, endocrine glands, kidneys and heart) of the body. Those output signals are important to synchronize the circadian rhythm with peripheral activities, by regulating the expression of specific genes, mainly *CLOCK* and *BMAL1* (elegantly reviewed in [69]), which are involved in many physiological functions, such as regulation of blood pressure, glomerular filtration rate and glucose metabolism [70]. Peripheral clocks, in turn, convey signals (hormones, cytokines) back to the central clock [69]. This entangled communication network between circadian clocks is involved in many physiological functions and it leads to integrated rhythmic control of metabolic and behavioral processes such as sleep cycle, hunger and body temperature. Environmental factors including macronutrient composition, meal timing, physical activity and light exposure, are considered external signals which might interfere with the circadian clocks. Emerging evidence suggests that disruption of this temporal synchronization between circadian clocks and environmental stimuli may lead to the development of obesity and its comorbidities [6,69,71,72,73] (Figure 2).

Indeed, mutations of genes involved in clock regulation are associated with overweight/obesity in humans [74,75,76]. Sleep pattern is an important factor for RMR [77,78]. In subjects with disruption of the sleep cycle due to conditions such as late sleeping, jet lag and night-shift work, a reduction in RMR was observed [7,79,80]. Lean healthy volunteers with 1-day shift and 2-day nightshift schedules showed increased total daily EE on the transition day to nightshift 1 and reduced total daily EE on nightshifts 2 and 3 [35]. The reduction in 24-h EE during these nightshifts would lead to positive energy balance and weight gain. The effects of the sleep pattern on body weight might be driven by changes in eating habits and meal timing. 

Recent studies have suggested that energy intake occurring mainly earlier during the day promotes long-term weight control. In a cross-sectional study, among 239 participants, those who consumed ≥33% (versus <33%) of their daily energy intake at 12.00 h were less likely to be overweight/obese, whereas those who consumed ≥33% of daily energy intake in the evening were two-fold more likely to develop overweight/obesity [81]. In a cohort study of 4243 subjects recruited from 2008–2010 and followed-up until 2012, a decreased risk of weight gain at follow-up was associated with greater energy intake during lunchtime [82] compared to dinner. Likewise, 1425 normal weight subjects who had the majority of their daily intake at dinner (~50%) showed an increased risk to develop obesity after 6 years follow up time [83] compared to those subjects who ate more at breakfast or lunch. In a randomized weight-loss study, women with overweight or obesity who were fed with a greater amount of energy at breakfast (700 kcal breakfast, 500 kcal lunch, 200 kcal dinner) showed greater weight loss compared to those who consumed higher energy intake at dinner (200 kcal breakfast, 500 kcal lunch, 700 kcal dinner) for 12 weeks [84]. In 279 subjects with obesity who underwent bariatric surgery and consumed late lunch (after 1500 h), a lower weight-loss response was observed compared to those individuals who had lunch at normal times (before 1500 h), after a 6-year follow-up time [85]. A recent study on 83 subjects with obesity reported that later meal and sleep timing were associated with an increase in the percentage of body fat [86]. Overall, these studies indicate that a larger total energy intake later during the day or at nighttime increases the risk to become overweight or develop obesity. This association appears to be stronger for carbohydrate and protein intakes than for fat intake [87,88]. However, additional studies showed no association between greater evening intake and body mass index (BMI) [89,90,91,92,93], while another one showed an inverse association [94]. A recent metanalysis of observational studies could not demonstrate a significant association between BMI and greater evening intake [95]. Due to the high heterogeneity and the risk of bias, particularly in observational trials, further controlled intervention trials are needed to provide definitive conclusions regarding the effect of higher energy intake at dinner on body weight change.

The question arises as to whether the putative effects of meal timing on body weight are related to changes in EE. Several studies have shown that the main components of 24-h EE such as RMR, PA and TEF might be affected by disruption of the circadian rhythm [96,97]. In healthy men and women, RMR was 6% lower in the early morning compared to the RMR at noon [98], although the latter could have been influenced by residual TEF from breakfast. At variance, following a one-week intervention, women fed with late lunch (1630 h) had a decrease in RMR as well as in carbohydrate oxidation, compared to early lunch (1300 h) subjects [99]. Many studies, however, failed to demonstrate the effect of meal timing on EE. In 10 young adults, no difference in 24-h EE was observed when normal meal time (1900 h) was compared to late evening meal (2230 h), although postprandial increase in EE shifted to later at night in those subjects who ate later at night [100]. The RMR measured after overnight fasting did not change when subjects eating breakfast were compared to those skipping breakfast, either in lean subjects [101] and in subjects with obesity [102]. Similarly, no difference in RMR was observed in a cross-over design study in which 10 lean female volunteers consumed breakfast cereal between 0700 h and 0800 h for 2 weeks, and for another 2 weeks had their first meal between 1030 h and 1100 h [103]. 

TEF accounts for ~10% of 24-h EE and it is the component of 24-h EE most likely to be affected by the circadian rhythm [63,104]. Studies have examined the effect of circadian and diurnal variation on TEF, without consistent results. Nine young men fed with the same meal at three different times of the day (0900, 1700 and 0100 nighttime) showed lower TEF following the night meal, compared to TEF after morning and afternoon meals [104]. Similarly, 14 healthy subjects showed a reduction in TEF of 4% after late meals during the first night shift compared to dinner at normal times [35]. In healthy volunteers in a controlled laboratory setting, including fixed energy intake, fixed sleep/wake cycles and no possibility to exercise [105], TEF was lower (44%) following a meal given at normal dinner time (2000 h) compared to the one provided at early morning. Consistently, another study showed significantly higher values of energy expenditure after the morning meal compared to values measured when the same meal was consumed in the evening [106].

A recent trial in 16 men fed with low-calorie breakfast and high-calorie dinner or high-calorie breakfast and low-calorie dinner in a crossover design showed that the TEF was 2.5-fold higher in the morning than in the evening independent of the calorie content of meals [107]. It has been recently shown that the activity of brown adipose tissue (BAT), which plays a role in regulating whole-body EE and substrate metabolism [108], was higher in the morning than in the evening. Interestingly, TEF after breakfast in the high-BAT activity group was significantly higher than that in the low-BAT group [109], suggesting that brown adipose tissue may play a role in affecting TEF after breakfast more than after dinner. These results could shed light on the possible association between eating habits and the development of obesity, since skipping breakfast and/or eating more at night might lead to an increase in body fat accumulation due to the reduction of TEF and daily energy expenditure.

On the other hand, TEF was not different in 10 lean young men who consumed meals in the morning compared to the afternoon [110] and no changes were observed in 11 subjects with obesity following calorically equivalent meals provided at 0730 h and 1200 h [111]. In 10 young adults who were fed with identical meals at normal (1900 h) or late (2230 h) evening meal, no differences in TEF were observed [100]. The discrepancy among these findings might be due to differences in study protocols, including less marked oscillations in the short daytime periods (morning vs. afternoon, 4–5 h) compared to daytime period morning vs. night (10–12 h), as well as shorter duration of EE measurements to fully capture the TEF in the hours following meal consumption before metabolic rate returns to the RMR baseline level. In any case, it should be considered that an increase in TEF, followed by weight loss, is likely to be compensated by a reduction in TEE [112]. Therefore, the clinical impact of a relatively small increase in TEF on weight change and obesity prevention may be of limited relevance.

The macronutrient composition of food items might affect the magnitude of TEF due to the alterations in substrate oxidation rates [7]. Protein oxidation rate measured in seven healthy males who consumed standard meals over a 30-h period showed circadian oscillation with the lowest urinary urea concentration at 2300–0200 h, and peak at 0800–1100, whereas no variations in carbohydrate and fat oxidation rates over 24 h were observed [97]. In contrast, in 10 healthy adult volunteers fed a high-fat diet during a desynchrony protocol, the carbohydrate oxidation rate reached the peak in the morning and the nadir during the evening, whereas lipid oxidation rate behaved in the opposite manner (peak in the evening, nadir in the morning) [113]. In another comparable study, no variations were observed in lipid oxidation rate, but a 10% reduction in carbohydrate oxidation rate was observed in the evening compared to morning in 14 healthy volunteers [105]. Seven males who consumed two isocaloric diets (high fat or high carbohydrate) in a crossover design showed a diurnal pattern in fat oxidation (nadir at 0800 h) with the high-fat diet, which was not observed for carbohydrate or protein oxidation [114]. Recently, a cross-over study evaluating the meal timing effects on 24-h EE in 11 adults with overweight showed an increase in TEF driven by an increase in fat oxidation in those volunteers who practiced early time-restricted feeding (from 8 am to 2 pm) compared to the control schedule (from 8 am to 8 pm) [115]. Although it is still a matter of discussion whether differences in substrate oxidation rates may affect the 24-h EE, current evidence suggests that meal timing and macronutrient composition of evening meals might have a role in substrate oxidation efficiency during the night. As a limitation, it should be highlighted that several studies did not have measurements of 24-h EE, and in many instances are based on self-reported questionnaires for food intake and body weight.

## 4. Conclusions

Emerging evidence suggests that higher caloric intake in the morning than later during the day is associated with reduced susceptibility to weight gain, although further controlled intervention trials are needed to provide definitive conclusions. TEF might play a role in the regulation of body weight based on the circadian rhythm. Daytime-loaded energy intake seems to be associated with higher TEE and TEF compared to later hours. Thus, a late daily energy load might favor a positive energy balance which, in turn, would promote weight gain. The study of circadian rhythm and/or specific meal and sleep timing patterns might play a role in identifying subjects more resistant or more prone to gaining weight. The alignment of meal and sleep timing to the normal circadian rhythm might become an important strategy aimed at controlling body weight and sustaining weight loss following any type of dietary intervention.

## Data Availability

Not applicable.

## Abbreviations

EI	energy intake
EE	energy expenditure
PA	physical activity
RMR	resting metabolic rate
TEF	thermic effect of food
